# Depressive symptoms moderate functional connectivity within the emotional brain in chronic pain

**DOI:** 10.1192/bjo.2023.61

**Published:** 2023-05-10

**Authors:** Yann Quidé, Nell Norman-Nott, Negin Hesam-Shariati, James H. McAuley, Sylvia M. Gustin

**Affiliations:** NeuroRecovery Research Hub, School of Psychology, The University of New South Wales, Sydney, New South Wales, Australia; and Centre for Pain IMPACT, Neuroscience Research Australia, Randwick, New South Wales, Australia; Centre for Pain IMPACT, Neuroscience Research Australia, Randwick, New South Wales, Australia; and School of Health Sciences, The University of New South Wales, Sydney, New South Wales, Australia

**Keywords:** Chronic pain, depression, functional connectivity, emotion, mood

## Abstract

**Background:**

Depressive symptoms are often comorbid with chronic pain. These conditions share aberrant emotion processing and regulation, as well as having common brain networks. However, the relationship between depressive symptoms and chronic pain and the effects on emotional brain function are unclear.

**Aims:**

The present study aimed to disentangle the effects of chronic pain and depressive symptoms on functional connectivity between regions implicated in both these conditions.

**Method:**

Twenty-six individuals with chronic pain (referred to as the pain group) and 32 healthy controls underwent resting-state functional magnetic resonance imaging and completed the Beck Depression Inventory. Main effects of group, depressive symptoms (total severity score) and their interaction on the functional connectivity of three seed regions (the left and right amygdalae and the medial prefrontal cortex; mPFC) with the rest of the brain were evaluated. In cases of significant interaction, moderation analyses were conducted.

**Results:**

The group × depressive symptoms interaction was significantly associated with changes in connectivity between the right amygdala and the mPFC (family-wise error-corrected *P*-threshold (pFWEc = 0.008). In the moderation analysis, the pain group showed weaker connectivity between these regions at lower levels of depressive symptoms (*P* = 0.020), and stronger connectivity at higher levels of depressive symptoms (*P* = 0.003), compared with the healthy controls. In addition, the strength of connectivity decreased in the healthy controls (*P* = 0.005) and increased in the pain group (*P* = 0.014) as the severity of depressive symptoms increased.

**Conclusions:**

Depressive symptoms moderate the impact of chronic pain on emotional brain function, with potential implications for the choice of treatment for chronic pain.

Chronic pain is defined as persistent experience of pain for over a period of 3 months, beyond normal healing time after injury or illness.^1^ Along with anticonvulsants (gabapentin, pregabalin), antidepressants, including serotonin noradrenaline reuptake inhibitors and tricyclic antidepressants, are considered to be first-line recommendations by the International Association for the Study of Pain.^2^ This may be owing to, at least in part, aberrant affective and mood processing and regulation being common comorbid consequences of chronic pain.^3^ Although depression or depressive symptoms are reported in around 60% of individuals with chronic pain,^3^ the relationship between chronic pain and depression on brain function is not well characterised. It remains unclear how both conditions affect shared brain phenotypes, especially the emotional brain,^4^ and whether the impact of depression is additive to the features reported in chronic pain. The present study aimed to determine the independent and interactive effects of chronic pain and depressive symptoms on emotional brain function.

Transition to chronic pain is associated with changes in connectivity within and between brain networks.^5^ In particular, whereas stronger connectivity between the medial prefrontal cortex (mPFC) and the nucleus accumbens^6^ is associated with the chronification of pain, a recent study reported that weaker nucleus accumbens–mPFC functional connectivity was associated with higher levels of anxiety in patients with chronic fibromyalgia.^7^ Graph theoretical approaches were used to further refine this model, showing increased density of connectivity of a network including the dorsal mPFC, nucleus accumbens, and amygdala in individuals transitioning to chronic pain compared with recovering individuals.^8^ More generally, subcortical (amygdala, hippocampus, nucleus accumbens) and cortical (mPFC) regions involved in emotion processing, affect regulation and memory have been consistently associated with pain experience.^9^

Individuals diagnosed with major depressive disorder also show aberrant connectivity between these regions. Compared with healthy individuals, people with major depressive disorder show decreased functional connectivity between the mPFC and posterior cingulate cortex (PCC)/precuneus nodes,^10^ as well as between the amygdala and the mPFC.^11^ In addition, further evidence indicates that compared with healthy individuals, people with major depressive disorder have weak positive connectivity between the nucleus accumbens and the mPFC and strong negative connectivity between the amygdala and the mPFC.^12^ Although depressive symptoms and chronic pain often co-occur and may share aberrant neurochemical balance and brain networks,^13^ it is also possible that the severity of depressive symptoms may moderate the changes in functional connectivity between regions involved in emotion processing and/or regulation associated with chronic pain.

Only few studies have investigated the functional effects of depressive symptoms and chronic pain in human. A recent animal study revealed the importance of a neural network involving the dorsal raphe nucleus and amygdala,^14^ confirming the key role of the amygdala in pain–depression comorbidity in humans. In a recent meta-analysis of five studies,^15^ right amygdalar/parahippocampal dysfunction was evident in people with a primary diagnosis of pain disorder with concomitant depression. Another meta-analysis showed no evidence for shared functional connectivity among large-scale networks in people with major depressive disorder and chronic pain; however, this study did not investigate the concomitant presence of these disorders.^16^ Although chronic pain and depressive symptoms share abnormal patterns of brain activation in the emotional brain, the relationship of patterns of functional connectivity among these regions remains unclear.

In this study, we set out to determine whether the severity of reported depressive symptoms moderated changes in functional connectivity of regions involved in emotion processing (amygdala) and regulation (mPFC) among people suffering from chronic pain and healthy individuals. In particular, consistent with patterns observed in major depressive disorder,^11^ we expected that increasing levels of depressive symptoms would be associated with weaker amygdala–mPFC connectivity in healthy participants and with stronger connectivity in people with chronic pain.^8^ In addition, we expected that depressive symptoms would be associated with aberrant connectivity between the mPFC and the PCC/precuneus, independently of the group.^10^

## Method

All participants were volunteers who provided informed consent according to procedures approved by the Human Research Ethics committees of the University of New South Wales (HC15206), the University of Sydney (HREC06287) and Northern Sydney Local Health District (1102-066M). The authors assert that all procedures contributing to this work comply with the ethical standards of the relevant national and institutional committees on human experimentation and with the Helsinki Declaration of 1975, as revised in 2008.

### Participants

Participants comprised 26 individuals suffering from chronic pain (together referred to as the pain group), including spinal cord injury neuropathic pain (*n* = 11), painful temporomandibular disorder (TMD, *n* = 4), trigeminal neuropathic pain (TNP, *n* = 10) and postherpetic neuralgia (*n* = 1), and 32 pain-free healthy controls (see Table 1 for details). Inclusion criteria for all participants were age over 18 years old with no known diagnosis of psychiatric disorder, especially major depressive disorder; no participant was followed by a psychiatrist or clinical psychologist at the time of recruitment. General exclusion criteria included heart pacemaker, metal implants, intrauterine contraceptive device, insulin pump, infusion devices, hearing-disease, claustrophobia, pregnancy, a history of stroke, multiple sclerosis or Parkinson's disease. All participants in the pain group experienced chronic pain, that is, pain for longer than 3 months.^17^ Neuropathic pain after spinal cord injury was diagnosed according to the International Association for the Study of Pain Spinal Cord Injury Pain Taxonomy.^18^ All people with spinal cord injury suffered from a complete paraplegia with continuous burning and/or shooting pain in areas of sensorimotor loss. Painful TMD is characterised by ongoing musculoskeletal facial pain as assessed using the research diagnostic criteria for TMD.^19^ TNP and postherpetic neuralgia, which are both characterised by continuous dull neuropathic facial pain with sharp exacerbations, were diagnosed using the Liverpool Criteria.^20^

**Table 1:** Sociodemographic and clinical characteristics of the studied cohort

	HC (*N* = 32)	Pain (*N* = 26)	Statistics Welch/*t*/χ^2^	d.f.	*P*-value
Age in years, mean (s.d.) [range]	45.29 (15.78) [22.19–71.76]	51.64 (10.28) [23.77–79.18]	1.846	53.67	0.070
Sex, *n* (female/male)	16/16	15/11	0.341	1	0.559
BDI total score (s.d.) [range]^a^	5.09 (4.63) [0–19]	13.30 (2.67) [0–34]	**2.325**	**38.30**	**0.025**
No or minimal depression (BDI score ≤ 9), *N* (%)	29 (91%)	17 (65%)			
Mild depression (10 ≤ BDI score ≤ 18), *N* (%)	2 (6%)	6 (23%)			
Moderate depression (19 ≤ BDI score ≤ 29), *N* (%)	1 (3%)	2 (8%)			
Severe depression (BDI score ≥ 30), *N* (%)	0	1 (4%)			
STAI state, mean (s.d.) [range]	27.88 (8.40) [20–54]	31.42 (9.30) [14–46]	1.525	56	0.133
STAI trait, mean (s.d.) [range]^b^	31.16 (8.19) [20–50]	36.04 (10.92) [19–53]	1.925	55	0.059
Pain duration in years, mean (s.d.) [range]	−	14.04 (12.80) [2–48]	−	−	−
VAS pain diary, mean (s.d.) [range]	−	3.90 (2.04) [0.2–8.8]	−	−	−
VAS scan pain, mean (s.d.) [range]	−	3.03 (1.87) [0–6.6]	−	−	−
Medication, *n* (%)	−	11 (42%)	−	−	−
Amitriptyline, *n* (%)	−	1 (4%)	−	−	−
Gabapentin, *n* (%)	−	2 (8%)	−	−	−
Pregabalin, *n* (%)	−	3 (12%)	−	−	−
Desvenlafaxine (SNRI), *n* (%)		1 (4%)			
Paracetamol PRN, *n* (%)	−	1 (4%)	−	−	−
Pregabalin + oxycodone, *n* (%)	−	1 (4%)	−	−	−
Oxycodone + paracetamol p.r.n., *n* (%)	−	1 (4%)	−	−	−
Codeine + paracetamol + ibuprofen p.r.n., *n* (%)	−	1 (4%)	−	−	−
Pain location					
Left, *n* (%)	−	3 (12%)	−	−	−
Right, *n* (%)	−	4 (15%)	−	−	−
Bilateral, *n* (%)	−	19 (73%)	−	−	−
Scanning sites (NeuRA/SVH)	16/16	12/14	0.085	1	0.798

HC, healthy controls; pain, individuals with chronic pain; BDI, Beck Depression Inventory; STAI, State-Trait Anxiety Inventory; VAS, visual analogue scale; SNRI: selective serotonin and norepinephrine reuptake inhibitor; NeuRA, Neuroscience Research Australia; SVH, St Vincent's Hospital Sydney.

a. Significant group differences are shown in bold.

b. Missing value for one HC participant.

### Assessments

Severity of depressive symptoms was measured using the sum of all 21 items from the Beck Depression Inventory (BDI; total score ranging from 0 to 63).^21^ Cut-off scores for estimation of the severity of depressive symptoms are: 0 to 9 for no or minimal depression, 10 to 18 for mild depression, 19 to 29 for moderate depression and 30 to 63 for severe depression. The BDI is a reliable measure of depressive symptoms in chronic pain populations.^22^ Severity of state and trait anxiety were assessed using the two 20-item subscales (scores ranging from 20 to 80) from the State-Trait Anxiety Inventory (STAI).^23^ Pain intensity was measured in the pain group only using a visual analogue scale (VAS); participants reported their experienced levels of pain on a 10-cm horizontal ruler, with ‘no pain’ being at the beginning of the ruler (0 cm mark) and ‘worst pain imaginable’ at the other extremity (10 cm mark) three times a day (morning, noon and evening). Two measures of pain intensity were collected using the VAS: the ‘pain diary’ consisted of an average measure of pain intensity for 7 days prior to the scanning day, and the ‘scan pain’ was a retrospective measure of pain intensity while the participant was lying in the scanner. Duration of pain, pain location (left, right or both sides of the body) and medications used were also recorded (Table 1).

### Magnetic resonance imaging (MRI)

Imaging data were acquired for each participant on two Philips 3T Achieva TX scanners (Philips Healthcare, The Netherlands) housed at Neuroscience Research Australia (Randwick, New South Wales, Australia; healthy controls: *n* = 16, pain: *n* = 12) or at St Vincent's Hospital (Darlinghurst, New South Wales, Australia; healthy controls: *n* = 16, pain: *n* = 14). Both scanners were equipped with eight-channel head-coils and used the same acquisition parameters to collect a three-dimensional T1-weighted structural image covering the entire brain: repetition time = 5.6 ms, echo time = 2.5 ms, field of view = 250 × 250 × 174 mm, matrix 288 × 288, 200 sagittal slices, flip angle = 8°, voxel size 0.9 × 0.9 × 0.9 mm. Furthermore, 180 whole-brain T2-weighted echo-planar images (repetition time = 2000 ms, echo time = 30 ms, field of view 240 × 140 × 240 mm, matrix 80 × 78, 35 slices, slice thickness = 4 mm, flip angle = 90°, voxel size 3 × 3 × 4 mm) were acquired, with participants asked to close their eyes and let their mind wander without falling asleep.

Pre-processing was performed with the CONN toolbox (version 20b; https://sites.google.com/view/conn/)^24^ for SPM12 (version 7771, Wellcome Department of Cognitive Neurology, University College London, UK; https://www.fil.ion.ucl.ac.uk/spm/) in MATLAB r2021a (MathWorks Inc., Sherborn, Massachusetts, USA). In addition to automatically discarded dummy scans, the first five acquisitions (10 s) for each subject were tagged as ‘outliers’ during the CONN outlier detection step, and their effects were removed during the denoising step. Pre-processing steps included realignment and unwarping, identification of outlier slices (ART toolbox, with movement translation threshold: 0.9 mm and signal intensity threshold *z* = 5), segmentation and normalisation of the functional and structural images, and smoothing with an 8-mm Gaussian kernel. Functional and anatomical data were resampled to a 180 × 216 × 180 mm bounding box (CONN default settings). In particular, functional images were resampled with 2-mm isotropic voxels, whereas structural data were resampled with 1 mm, using fourth-order spline interpolation. Temporal band-pass filtering (0.008 < *f* < 0.09) was applied to reduce the effects of low-frequency drift and high-frequency noise. As per the default settings of the toolbox, physiological and other potential sources of noise (white matter, cerebrospinal fluid) were estimated using a component-based noise correction method (CompCor)^25^ and regressed out along with movement-related effects, constant and first-order linear session effects, and scrubbing covariates. Only participants with fewer than 18 volumes (10% of the total number of acquisitions) identified as outliers by ART (other than the initial five volumes) were included in the analyses.

### Seed-based connectivity

Seed-based functional connectivity maps (seed-to-voxel bivariate correlations) were derived for all participants and all regions of interest available within the CONN toolbox. The mPFC seed region was from the CONN network cortical region of interest atlas,^24^ derived from an independent component analysis performed on 497 participants from the Human Connectome Project data-set (10-mm-diameter spheres around Montreal Neurological Institute (MNI) coordinates [1, 55, −3]), and the left and right amygdala seed regions were from the FSL Harvard–Oxford maximum likelihood subcortical atlas (HarvardOxford-sub-maxprob-thr25-1 mm.nii). Individual functional connectivity maps (bivariate correlations) for these seed regions were Fisher *r*-to-*z* transformed and exported for further group-level analyses.

### Harmonisation

Before being entered into second-level (group) analyses, individual first-level connectivity maps (for each seed region) were harmonised using the Python-based neuroHarmonize tools (https://github.com/rpomponio/neuroHarmonize).^26^ Briefly, this approach uses empirical Bayes methods derived from the ComBat R package^27^ to adjust whole-brain statistical maps for variation associated with scanning location in multisite MRI studies. Age, sex and group (healthy controls or pain) were modelled as covariates during harmonisation to ensure neuroHarmonize did not remove the variance associated with these variables.

### Statistical analyses

Owing to the relatively small sample size, statistical analyses were performed using the Statistical Non-Parametric Mapping (SnPM13.1.08; http://www.nisox.org/Software/SnPM13/) toolbox for SPM12.^28^ This toolbox uses permutation tests that, unlike parametric statistics, do not rely on assumptions of normality and are therefore less likely to produce false positive results. Here, the SnPM13 toolbox was set to perform 10 000 permutations, and variance smoothing was not applied (set to [0,0,0]). A series of multiple linear regressions were performed to determine the main effects of group (healthy controls versus pain), severity of depressive symptoms (BDI total score) and their interaction (the product of group × mean-centred BDI total score) on patterns of seed-based connectivity (one model for each seed). Age and sex were added as covariates in all neuroimaging analyses. Whole-brain statistical significance was set at an uncorrected voxel-wise threshold of *P* < 0.001, to which family-wise error correction was applied to the cluster statistics (family-wise error-corrected *P*-threshold (pFWEc) < 0.05). An additional Bonferroni correction was applied to cluster statistics to account for the number of seed regions studied (pFWEc < 0.017).

In case of significant interaction (pFWEc < 0.017), the signal at the peak of each identified cluster was extracted, and moderation analyses were formally tested using the interactions package (version 1.1.5) in R (version 4.1.2) and RStudio (version 1.4.1717). Unlike mediation analyses, which identify and explain the mechanism or process an underlying observed relationship between an independent variable and a dependent variable via the inclusion of a third hypothetical variable, moderation analyses assume that the relationship between the independent variable (predictor) and the dependent variable (outcome) is dependent on the level of a third variable (moderator). Thus, unlike in mediation analyses for which it is recommended (but not mandatory), a direct association between the independent variables (here, the pain and healthy controls groups) and the dependent variables (here, brain connectivity) is not required for moderation analyses, as this relationship is likely to be dependent on variations in the moderator (here, BDI scores). First, we tested our *a priori* hypothesis that the severity of depressive symptoms would moderate the impact of chronic pain on functional connectivity. Second, for the sake of completeness, group was also tested as a moderator of the relationship between the severity of depressive symptoms and patterns of functional connectivity. In addition, within each model, the Davidson–McKinnon correction (HC3) was used to account for potential issues related to heteroskedasticity using the R package sandwich. Within each significant model, statistical significance was set at a threshold of *P* < 0.05.

## Results

### Participant characteristics

There were no significant differences in age, sex distribution, or levels of state or trait anxiety as measured by the STAI between the groups. However, the pain group reported higher levels of depressive symptoms (BDI total score) compared with the healthy controls group (Cohen's *d* = 0.65). In addition, scanning sites were similarly distributed across the groups [χ^2^(1*) =* 0.085, *P* = 0.798].

### Seed-based functional connectivity

#### Left amygdala

There were no significant main effects of group, depressive symptoms or their interaction on patterns of functional connectivity with the left amygdala seed region.

#### Right amygdala

**Fig. 1:**
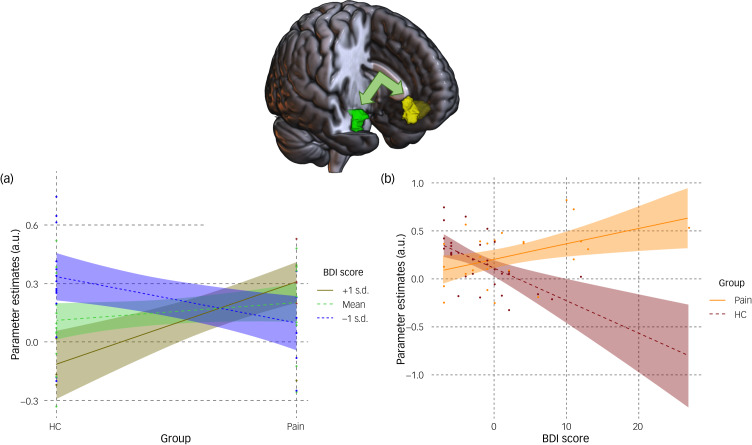
Association between group × depressive symptom severity interaction and functional connectivity between the right amygdala seed and the medial prefrontal cortex. Moderation analyses using (a) depressive symptoms and (b) groups as moderator indicate that: (a) compared with the healthy controls group (HC), the pain group showed weaker connectivity between the right amygdala (in green) and the medial prefrontal cortex (mPFC, in yellow) at low levels of depressive symptoms (blue dotted line) but stronger connectivity at higher levels of depressive symptoms (brown plain line); there was no group difference at average levels of depressive symptoms (green dashed line). (b) As levels of depressive symptoms increased, connectivity between the right amygdala and the mPFC decreased in the HC group (red dashed line) and increased in the pain group (orange plain line). The coloured band around each line represents the 95% confidence interval.

There was a significant association between the group × depressive symptom interaction and connectivity between the right amygdala seed region and a cluster in the mPFC (peak MNI coordinates [2, 42, −10], *k* = 677 voxels, *t*(52*)* = 4.41, pFWEc = 0.008). The moderation analysis was statistically significant (model statistics: adjusted *R*^2^ = 0.221, *F*(5,52) = 4.233, *P* = 0.003). When BDI score was entered as a moderator (Fig. 1(a)), connectivity between the right amygdala and the mPFC was weaker in the pain group compared with the healthy controls group at low levels of BDI scores (*b* = −0.236, s.e. = 0.098, *t* = −2.403, *P* = 0.020, 95% CI −0.433 to −0.039) but stronger at high levels of BDI scores (*b* = 0.421, s.e. = 0.134, *t* = 3.142, *P* = 0.003, 95% CI 0.152 to 0.689). There were no group differences in patterns of connectivity between the right amygdala and the mPFC at average BDI scores (*b* = 0.092, s.e. = 0.075, *t* = 1.225, *P* = 0.226, 95% CI −0.059 to 0.244). When group was entered as a moderator (Fig. 1(b)), the moderation analysis indicated that increasing BDI scores were associated with decreased connectivity between the right amygdala and the mPFC in the healthy controls group (*b* = −0.034, s.e. = 0.012, *t* = −2.907, *P* = 0.005, 95% CI −0.058 to −0.011) and with increased connectivity in the pain group (*b* = 0.016, s.e. = 0.006, *t* = 2.553, *P* = 0.014, 95% CI 0.003 to 0.028).

This was in the context of no significant effects of depressive symptoms (BDI total score) or group (healthy controls versus pain) on connectivity with the right amygdala seed.

#### Medial prefrontal cortex

There was a weak effect of group on connectivity between the mPFC seed and a cluster at the junction between the right inferior temporal gyrus and the right temporal pole (peak MNI coordinates [46, 4, −36], *k* = 312 voxels, *t*(52*)* = 4.11, pFWEc = 0.028), which did not survive the additional Bonferroni correction (pFWEc < 0.017). There were no significant effect of depressive symptoms or depressive symptoms × group interaction with connectivity between the mPFC and any other region in the brain.

### Exploratory correlation analyses

Exploratory bivariate Pearson's correlation analyses were conducted to rule out potential confounding effects of pain duration and pain intensity (using the pain diary measure) on levels of depression (BDI total score). However, there were no significant associations between pain duration (*r* = −0.035, *P* = 0.864) or pain intensity (*r* = 0.063, *P* = 0.760) and levels of depressive symptoms.

## Discussion

In the context of overall more severe depressive symptoms in the pain group compared with healthy controls, depressive symptoms moderated the impact of chronic pain on resting-state functional connectivity between regions critical for emotion processing (right amygdala) and regulation (mPFC). In particular, compared with controls, people with chronic pain showed weaker connectivity between the right amygdala seed region and the mPFC at low levels of depressive symptoms and stronger connectivity at high levels of depressive symptoms. In addition, as levels of depressive symptoms increased, connectivity between these regions decreased in healthy controls and increased in people with pain.

Partly consistent with our hypothesis, the relationship of the strength of resting-state functional connectivity between the right (but not left) amygdala and the mPFC with chronic pain (compared with healthy controls) was moderated by the severity of depressive symptoms reported: compared with controls, weaker connectivity was evident at lower levels of depressive symptoms, and stronger connectivity was evident at higher levels of depressive symptoms, in people with chronic pain. As a core node of the default mode network, the mPFC specialises in the treatment of affective stimuli and exerts inhibitory top-down control on amygdalar activity.^29^ Importantly, the mPFC is also a central hub for both cognitive and affective comorbid states often reported in chronic pain, including depression.^30^ In addition, increased severity of depressive symptoms in the healthy controls group was associated with decreasing amygdala–mPFC connectivity strength. This is consistent with previous reports of reduced connectivity between these regions in adults and adolescents with major depressive disorder,^11^ and with reduced top-down control over amygdala activity leading to the inability of the mPFC to downregulate negative affect.^31^ On the other hand, increasing severity of depressive symptoms was associated with increasing strength of connectivity in people with chronic pain. This suggests that the top-down regulation provided by the mPFC to the amygdala may be inefficient, reflecting potential mood-related maladaptive consolidation of aberrant affective information in this population.

Mechanisms by which depressive symptoms affect functional connectivity between the mPFC and amygdala in chronic pain are unclear but may be associated with changes (increases) in peripheral inflammation. Increased levels of peripheral inflammation are common in chronic pain and depression^32^ and are considered a common mediator in both conditions.^33^ Increased inflammation can be triggered by sustained exposure to psychosocial stressors, such as chronic pain,^34^ inducing the release of stress-related glucocorticoids (i.e. cortisol). Released cortisol will in turn trigger glia activation and cytokine production, which downregulate glutamate levels in the mPFC, as observed in both chronic pain^35^ and depression,^36^ and may result in inefficient mPFC top-down regulation. Results from the present study indicate that changes in functional connectivity between the mPFC and the amygdala are sensitive to depressive symptoms in individuals with chronic pain. Although these changes are different (in the opposite direction) in people with chronic pain compared with pain-free individuals, they may operate on a continuum, via common neurobiological pathways including chronic stress, inflammation and glutamate availability in the mPFC. This mechanistic explanation is plausible but remains speculative, and future large longitudinal studies integrating markers of inflammation, brain function and neurochemistry, as well as clinical and behavioural phenotypes, are necessary to better understand and confirm these mechanisms.

Contrary to our hypothesis, the connectivity between the mPFC seed and the PCC/precuneus was not associated with the severity of depressive symptoms. Importantly, our results provide some evidence that the connectivity between the mPFC and PCC/precuneus might not be sensitive to the severity of depressive symptoms but rather associated with a diagnosis of major depressive disorder.^10^ We also note that none of our participants was formally diagnosed with major depressive disorder, and that our study may have lacked enough statistical power to uncover more subtle effects. Future studies including a group of individuals with chronic pain and major depressive disorder are required to disentangle the changes in the default-mode network connectivity that are specific and common to chronic pain and depression.

Overall, the results indicate that depressive symptoms and emotional brain circuits are potential targets for interventions in chronic pain. For instance, repeated transcranial magnetic stimulation (rTMS) targeting the mPFC could help to normalise the top-down control of the mPFC on the amygdala, especially in people reporting higher levels of depression. Significant reduction of symptom severity in depressed individuals was reported after mediofrontal double cone coil stimulation of the anterior cingulate cortex, compared with that observed with typically prescribed rTMS of the left dorsolateral prefrontal cortex.^37^ Another study reported reduced depressive symptoms following stimulation of the right orbitofrontal cortex (at the AF8 site, defined according to the international 10–20 EEG system).^38^ It is important to note that both of these stimulation sites are close to but do not exactly match the location of our mPFC cluster. Efficacy of rTMS of the mPFC in reducing depressive symptoms and normalising emotional processing/regulation in people with chronic pain should be considered for future clinical trials. Normalisation of emotion/affect dysregulation can reduce pain severity^39^ and could be key to preventing the development and maintenance of chronic pain. It is also important to note that people with different types of chronic pain (i.e. neuropathic and nociceptive) exhibit comparable negative affective–motivational and cognitive–evaluative states, including similar levels of depression.^40^ Thus, targeting emotional/affective processing areas such as the mPFC may be key to reducing both affective and physical suffering regardless of chronic pain type.

This study had several limitations. First, although the non-parametric imaging statistical approach used was appropriate and accounted for the sample size, the study's sample size was relatively small, preventing the identification of smaller effects. In addition, the present hypothesis-driven study only investigated functional connectivity from three seed regions. Future studies may include a larger number of regions not specifically involved in emotion processing and regulation to uncover how depressive symptoms may differently affect other resting-state networks in chronic pain and healthy controls. Second, people with chronic pain included in this study had different conditions, and pain was reported at various locations over the body, which may potentially have influenced patterns of brain function. Despite these limitations, the observed effects were strong and were present in a heterogeneous group of individuals with pain, indicating that they may represent a common feature across pain disorders. There is indeed no indication that the type or location of pain would differently affect brain regions involved in emotion processing and regulation. However, replication studies in larger clinical groups are required. Third, most individuals in clinical cases were using a variety of medications, mostly analgesics. Although the amounts of drugs taken by each individual were not controlled for and may have influenced brain function, they represent an ecological sample of what people with chronic pain generally use. In addition, as all models tested included healthy participants, it was not possible to include information on medication (type, dosages or numbers) to account for these confounding factors. Future studies are needed to explore heterogeneity in pain disorders, including pain types, location, or medication, as this would help to rule out the confounding effects of these factors on functional connectivity in people with chronic pain. Finally, future studies are warranted to understand the role of functional connectivity between the amygdala and mPFC in mediating the relationship between pain and depressive symptoms.

In conclusion, severity of depressive symptoms moderates resting-state functional connectivity between regions critical for emotional recognition (amygdala) and regulation (mPFC) in people with chronic pain and healthy controls. These results may have implications for the choice of treatment for chronic pain, in the context of reported depressive symptoms. In particular, future studies should consider testing the efficacy of rTMS of the mPFC in people with chronic pain. Targeting the mPFC may ameliorate both affective and physical suffering in this population.

## Data Availability

The data that support the findings of this study are available from the corresponding author upon reasonable request.
